# Association of Genetic Variants in *IL6* Gene (rs1800795) with the Concentration of Inflammatory Markers (IL-6, hs-CRP) and Superoxide Dismutase in the Blood of Patients with Acute Pancreatitis—Preliminary Findings

**DOI:** 10.3390/genes13020290

**Published:** 2022-02-01

**Authors:** Monika Ołdakowska, Milena Ściskalska, Marta Kepinska, Grzegorz Marek, Halina Milnerowicz

**Affiliations:** 1 Department of Pharmaceutical Biochemistry, Division of Biomedical and Environmental Analyses, Faculty of Pharmacy, Wroclaw Medical University, Borowska 211a, 50-556 Wroclaw, Poland; monika.oldakowska@umw.edu.pl (M.O.); marta.kepinska@umw.edu.pl (M.K.); 2 Second Department of General and Oncological Surgery, Faculty of Medicine, Wroclaw Medical University, Borowska 213, 50-556 Wroclaw, Poland; grzegorz.marek@umw.edu.pl; 3 Department of Biomedical and Environmental Analyses, Faculty of Pharmacy, Wroclaw Medical University, Borowska 211, 50-556 Wroclaw, Poland; halina.milnerowicz@umw.edu.pl

**Keywords:** acute pancreatitis, interleukin 6, superoxide dismutase, rs1800795

## Abstract

In the course of acute pancreatitis, interleukin-6 plays an important role as a mediator in the inflammatory response. The course of inflammatory disease is associated with intensive oxidative stress, which may activate transcription factors leading to gene-expression changes. Isoenzymes of superoxide dismutase are involved in the defense against free radicals. This study aimed to evaluate changes in IL-6 concentration and the concentration/activity of superoxide dismutase isoenzymes (SOD1, SOD2, and SOD3) in the blood of patients with acute pancreatitis (AP) in terms of rs1800795 polymorphism in the *IL6* gene. In the smoking AP patients group with the GC and GG genotypes, the plasma SOD1 concentration was significantly higher (*p* = 0.0146 and *p* = 0.0250, respectively) than in patients with CC genotype for SNP rs1800795 in the *IL6* gene. An increase in SOD1 concentration in erythrocytes of AP patients with GC genotypes was also demonstrated compared to the individuals from the group with GG genotype (*p* = 0.0408). Furthermore, a positive correlation between IL-6 and SOD1 concentrations in the plasma of AP patients with GC genotype for SNP rs1800795 was shown. These results indicate that SOD1 may play a protective role against oxidative damage induced by inflammation in the group of AP patients with GC genotype.

## 1. Introduction

Acute pancreatitis (AP) is a sudden acute inflammation of the pancreas associated with premature activation of pancreatic enzymes and self-digestion of the pancreas, consequently activating the inflammatory cascade [1]. AP often involves the peripancreatic tissues and organs during disease progression [2]. Due to the high mortality associated with AP complications, its early diagnosis and prognosis are crucial [1,3]. Although pancreatic enzymes such as lipase and amylase are considered relevant markers in AP diagnosis of AP, the assessment of the activity of these parameters is unable to determine the severity of the disease [4]. One of the good prognostic marker is interleukin-6 (IL-6) [5]. This pleiotropic cytokine is involved in the regulation of acute-phase reactions and plays a key role in generating immune responses. IL-6 also participates in the pathogenesis of β cell destruction [5,6]. Many studies have shown the significant role of IL-6 as an early indicator of AP severity [7,8]. IL-6, through gp130 protein, takes part in the activation of the Janus kinase/signal transducers and activators (JAK/STAT) signaling pathway [9]. The concentration of this cytokine increases earlier, and it is a more sensitive marker of severity of inflammation than C-reactive protein (CRP) [10,11]. The release of IL-6 from the inflamed pancreas tissue is associated with the dysfunction of this organ [8,9]. The serum IL-6 concentration is elevated significantly in severe and fatal cases with the acute pancreatitis, as well as in cases complicated with pulmonary and renal failure [12]. The pathophysiological role of IL-6 in the acute phase response is due to its extensive pro-inflammatory effects on various cells and the induction of hepatic acute-phase protein production [5,13].

The human *IL6* gene contains five exons and four introns. This gene is located in position 7p15-21 [14,15]. The *IL6* gene takes part in the regulation of inflammatory pathways [16]. The presence of single nucleotide polymorphisms (SNP) rs1800795 (174G>C) in the promoter of the *IL6* gene was suggested to functionally affect the *IL6* gene promoter activity and thus cause an increase in IL-6 concentration [17,18]. Furthermore, it was shown that the CC genotype for rs1800795 in the *IL6* gene was associated with an increase in IL-6 and hs-CRP concentrations and thus an increased inflammatory response [19]. On this basis, it can be assumed that this genetic variation can be used as a genetic marker in inflammatory diseases such as acute pancreatitis.

It was demonstrated that the superoxide dismutase (SOD) isoenzymes could play a potential role as inflammatory inhibitors [20,21]. SODs are recognized as the first line of defense against oxidative stress due to their ability to neutralize reactive oxygen species (ROS) with pro-inflammatory potential, which can induce tissue damage [22,23]. ROS neutralization is based on the catalysis of the dismutation of the superoxide radical anion (O_2_^•−^) to molecular oxygen and hydrogen peroxide (H_2_O_2_) [24]. The SOD family presents three forms: intracellular copper-zinc SOD (cytosolic Cu/Zn-SOD, SOD1), mitochondrial SOD (Mn-SOD, SOD2), and extracellular copper-zinc SOD (EC-SOD, Cu/Zn-SOD SOD3) [25]. The participation of SOD isoenzymes in the course of pancreatitis was confirmed by increasing the expression of SODs in acinar cells of the pancreas [26]. Additionally, this enzyme is involved in protecting the cells against harmful effects of environmental factors with pro-oxidative potential, such as tobacco smoke exposure [27], which is recognized as a major risk factor of acute pancreatitis. 

This study was aimed to assess the relationship between genetic variants of polymorphism rs1800795 in the *IL6* gene and the concentration of inflammatory markers (IL-6 and hs-CRP) and the concentrations and activities of SOD isoenzymes in the group of AP patients. This study also investigated whether polymorphism rs1800795 may be associated with an increased risk of acute pancreatitis. It also assessed the impact of exposure to xenobiotics from tobacco smoke on the concentration of inflammatory markers, the concentration of SOD isoenzymes, and total SOD activity. This study can contribute to better understanding the molecular mechanism of the inflammatory process and antioxidant defense in the course of acute pancreatitis.

## 2. Materials and Methods

### 2.1. Subject

The study group consisted of 40 patients with acute pancreatitis (17 non-smokers and 23 smokers) aged 50.2 ± 15.4 (BMI: 25.0 ± 2.5 kg/m^2^), hospitalized in the Second Department of General and Oncological Surgery, Wroclaw Medical University. The group of healthy subjects was comprised of 51 volunteers (26 non-smokers and 25 smokers) aged 46.3 ± 8.5 (BMI: 23.0 ± 1.8 kg/m^2^). The study was conducted in accordance with the Declaration of Helsinki and approved by the Bioethics Committee of the Wroclaw Medical University (No.: 529/2018 and KB: 215/2020). 

All study participants completed a lifestyle questionnaire in which they provided the information about their health, diseases, diets, and drug and dietary supplement use. The questionnaire also included information about smoking history. 

Exposure to tobacco smoke xenobiotics was assessed by measuring the concentration of cotinine (a metabolite of nicotine). When the cotinine concentration was below 10 ng/mL, the participants were included to the non-smoking group, while in the case of a cotinine concentration above 10 ng/mL, the participants were qualified as smokers.

**Figure 1:**
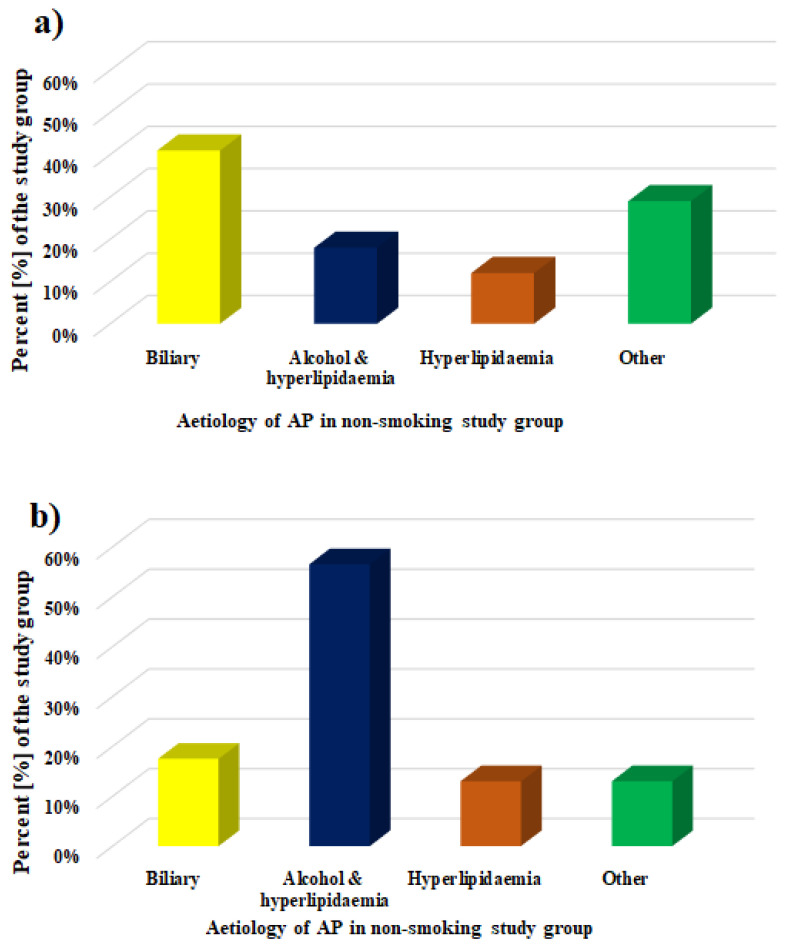
Etiology of AP in the group of (**a**) non-smoking and (**b**) smoking patients. AP—acute pancreatitis.

The patients were classified to the AP patients group based on a clinical interview, epigastric pain with touch sensitivity, and laboratory tests and imaging tests (computed tomography, magnetic resonance imaging, and ultrasound). Specific criteria for the inclusion of the patients were described earlier [28,29]. In cases when patients experienced abdominal pain typical of AP symptoms and a threefold increase in serum lipase or amylase (above the upper limit of normal), no imaging studies were performed to diagnose acute pancreatitis. In patients who did not experience abdominal pain typical for AP or when serum lipase or amylase were less than threefold of the upper limit of normal or when their diagnosis was uncertain, in order to diagnose acute pancreatitis and rule out other causes of acute abdominal pain, the abdominal cavity was imaged using computed tomography with contrast. Whereas, in patients with severe contrast allergy or renal failure, abdominal magnetic resonance imaging without gadolinium was used. The severity of the disease was determined according to the Atlanta classification. Ultimately, the study enrolled non-organ-failing patients, without local complications (qualified as mild AP patients). The exclusion criteria were all comorbidities such as cardiovascular diseases, liver diseases, cancer, diabetes, arthritis, and ongoing inflammatory states other than AP. In addition, patients were excluded from the study in cases when they were taking more than two types of medication regardless of their mechanism of action. The clinical characteristics of AP patients and the etiology of AP are presented in Table 1 and Figure 1, respectively. 

The comparison group consisted of healthy volunteers qualified to the study based on a physical examination by primary health care physicians, interviews, laboratory tests (morphology, lipid profile, and parameters describing glucose metabolism and parameters assessing liver and kidney dysfunction) and a questionnaire evaluation. The exclusion criteria included inflammatory and metabolic diseases, cardiovascular diseases, tumor diseases, and taking the medications and dietary supplements as described earlier [28,29].

### 2.2. Material

The whole blood was collected from the ulnar vein from patients up to 24 h from the onset of the first symptoms of acute pancreatitis. The blood from healthy subjects were obtained from the biobank of the Polish Center for Technology Development (Wroclaw, Poland). The material for the study were serum, plasma, and erythrocyte lysate. In order to obtain the serum, the blood was gathered in trace-element-free tubes with a serum clotting activator in accordance with the standard procedure (cat. No.: 368815, Becton Dickinson, Germany). To generate complete thrombosis, blood samples were left at 25 °C, and then they were centrifuged (1200× *g*/20 min). The plasma and erythrocytes were acquired by drawing whole blood in tubes containing heparin (Cat. No. 368886, Becton Dickinson) and EDTA (Cat. No. 367864, Becton Dickinson, Germany) and centrifuged (2500× *g*/15 min) to separate plasma and buffy coat from erythrocytes. An erythrocyte lysate was obtained by twice washing the erythrocytes 1:1 with 0.9% NaCl followed by a 1:1.4 dilution with ice-cold double-distilled water. The separated material was stored in sealed tubes (cat. no.: 0030102.002, Eppendorf, Germany) at −25 °C until analysis. DNA extracted from the buffy coat, contained in EDTA tubes, was stored at −80 °C.

### 2.3. Methods

The cotinine concentration in serum was assayed with the Cotinine ELISA kit (cat. No.: EIA-3242, DRG International, Inc., Springfield, NJ, USA).

The concentration of interleukin 6 (IL-6) in plasma was assayed with the ELISA kit (cat. no.: DY206-05, R&D Systems, USA).

The concentration of high-sensitivity CRP (hs-CRP) in serum was measured by the turbidimetric method with the C-reactive protein hs test (cat. no.: 31927, Biosystems, Spain).

The concentrations of intracellular copper-zinc superoxide dismutase (SOD1), manganese superoxide dismutase (SOD2), and extracellular superoxide dismutase (SOD3) were measured in plasma and erythrocyte lysate with ELISA kit (SOD1—cat. no.: BMS222, Thermofisher, Waltham, MA, USA; SOD2—cat. no.: EKU07502, Biomatik, Wilmington, DE, USA; SOD3—cat. no.: EKU07504, Biomatik, Wilmington, DE, USA). The concentrations of SOD isoenzymes in the erythrocyte lysate were expressed in ng/mg Hb.

The total SOD activity in plasma and erythrocyte lysate was determined by a commercial kit (cat. no.: 706002, Cayman Chemical, Ann Arbor, MI, USA). The concentrations of SOD isoenzymes in the erythrocyte lysate were expressed in ng/mg Hb.

### 2.4. Genotyping Analyses

The DNA was isolated directly from the buffy coat of platelet leukocytes with a kit (Syngen Blood/ Cell DNA Mini Kit, cat. no.: SY221012, Syngen Biotech, Wroclaw, Poland), following the manufacturer’s instructions. Then, the DNA purity and quantity were measured with a Thermo Scientific µDropPlate (cat. no.: N12391, Thermo Fisher Scientific, Waltham, MA, USA) at wavelengths of 260 and 280 nm. Next, the purity of the samples and the isolation efficiency were assessed by calculating the A280/A260 absorbance coefficient. The A280/A260 ratio was above 1.8, proving the purity of the isolated DNA. 

To determine rs1800795 polymorphisms in *IL6* gene (NC_000007.13:g.22766645C>G), the polymerase chain reaction and restriction fragment length polymorphism analysis (PCR-RFLP) was performed. The primers were designed with Primer-BLAST, based on the gene sequences from GenBank (National Center for Biotechnology Information). The PCR reactions in the *IL6* gene was performed on a 20.0 μL sample containing 20  ng of genomic DNA, 0.6 μL of primers (100 pmol of each forward and reverse prime), 12.8 μL PCR clean water, and 4 μL Gold Hot-Start PCR Mix (cat. no.: SY550231, Syngen). PCR reactions were conducted under the following conditions: the initial denaturation at 95 °C for 15 min, followed by 35 cycles of: denaturation at 95 °C within 40 s, annealing at 55 °C for 35 s, and elongation at 72 °C within 45 s. The final elongation step was performed at 72 °C for 15 min. The *IL6* gene primer sequence was 5′ TGACTTCAGCTTTACTCTTTGT 3′ (forward) and 5′ CTGATTGGAAACCTTATTAG 3′ (reverse) with a PCR product being 198 bp. The PCR products were digested with SfaNI restriction enzyme (cat. no. ER1621 Thermo Fischer, Waltham, MA, USA), and then the digested fragments were visualized in 2% agarose gel (cat. no.: SY521011 Syngen Biotech, Wroclaw, Poland) with Green DNA Gel Stain (cat. no.: SY521032, Syngen Biotech, Wroclaw, Poland). An undigested band of 198 bp was detected in GG homozygotes; digested DNA fragments of 140 and 58 bp were detected in CC homozygotes; and GC heterozygous genotypes resulted in three different bands (198, 140, and 58  bp). 

One of the exemplary electropherograms showing the products after digestion with the restriction enzyme in the group of AP patients and healthy subjects is given in Figure S1 (supplementary material paragraph).

### 2.5. Statistical Analysis

The data were presented as a mean ± standard deviation (SD) and a first quartile (Q1), a median, and a third quartile (Q3). The normality of the distribution was verified using the Shapiro–Wilk test. In the case of the normality of distribution, to assess a significant difference between two groups, the Student’s *t*-test was used. When the variables did not meet the conditions of the normal distribution, the nonparametric Mann–Whitney U test was used. The homogeneity of variance was analyzed using Levene’s test. To compare the parameters of the three groups (three genotypes), the ANOVA and Tukey’s post hoc test were used. The correlation was expressed by Spearman’s correlation coefficient (r). The differences in frequencies of genotypes were compared using a *χ*^2^ test and Fisher’s exact test. The univariate analysis was performed to assess the significance effect of polymorphism genotypes on the risk of disease, which was expressed as an odds ratios (OR) at 95% CI. A *p*-value < 0.05 was considered statistically significant. Data analyses were carried out using the Statistica Software Package, version 13.3 (Polish version; StatSoft, Krakow, Poland).

## 3. Results

### 3.1. The Concentration of Markers of Inflammation and SOD Isoenzymes and Total SOD Activity in the Group of AP Patients in Terms of Tobacco Smoke Exposure

In the blood of AP patients, IL-6 and hs-CRP concentrations were significantly higher both in non-smokers (*p* < 0.0001 for both parameters) and smokers (*p* = 0.0001 and *p* < 0.0001, respectively) compared to the non-smoking and smoking healthy subjects. In addition, a 1.5-fold increase in IL-6 concentration in the blood of smokers compared to non-smokers in the group of AP patients was shown (Table 2).

In the plasma of non-smoking and smoking AP patients, an almost three-fold increase in SOD1 concentration (*p* < 0.0001 for both subgroups) compared to healthy subjects was found. In addition, the plasma SOD2 concentration was two-fold lower in the group of non-smoking (*p* < 0.0001) and smoking (*p* < 0.0001) AP patients in comparison with healthy subjects. Whereas, in erythrocytes of non-smoking and smoking AP patients, a five-fold increase in SOD2 concentration (*p* = 0.0128 and *p* < 0.0001, respectively) compared to healthy subjects was observed. Furthermore, the plasma SOD3 concentration was significantly lower in the group of non-smoking (*p* = 0.0036) and smoking (*p* = 0.0149) AP patients in comparison with healthy subjects. Moreover, the total SOD activity in erythrocytes was significantly higher in the group of non-smoking (*p* < 0.0001) and smoking (*p* < 0.0001) AP patients compared to healthy subjects (Table 2). 

### 3.2. The Influence of the rs1800795 Polymorphism on the Concentration of Inflammatory Markers, in the Group of Non-Smoking and Smoking Healthy Subjects and AP Patients

In the group of AP patients, the GC genotype was found in 14 cases (35% of AP patients); the CC genotype was shown in 7 cases (17.5% of AP patients); and the GG genotype was found in 19 cases (47.5% of AP patients). In the group of the healthy subjects, the GC genotype was detected in 15 cases (2.5% of healthy subjects); the CC genotype was detected in 13 cases (25.5% of healthy subjects); and 23 cases with the GG genotype (45% of healthy subjects) were found. No differences in the frequency of occurrence of genotypes in the examined groups was shown (x^2^ = 0.4606, *p* = 0.8300). 

We analyzed the concentrations of inflammatory markers, the concentration of SOD isoenzymes, and total SOD activity in relation to SNP rs1800795 in *IL6* gene (NCBI Reference Sequence: NG_008689.1) in examined groups. Increased IL-6 and hs-CRP concentrations in the group of non-smoking AP patients with the GC (*p* = 0.0227, *p* = 0.0107), CC (*p* < 0.0001, *p* < 0.0001), and GG genotype (*p* = 0.0304, *p* = 0.0024) for rs1800795 in the *IL6* gene compared to healthy subjects with corresponding genotypes were demonstrated (Table 3). Additionally, increased IL-6 and hs-CRP concentrations in the group of smoking AP patients with the GC (*p* < 0.0001, *p* = 0.0294), CC (*p* < 0.0001, *p* = 0.0227), and GG (*p* = 0.0282, *p* = 0.0019) genotypes for rs1800795 in the *IL6* gene compared to smoking healthy subjects were found (Table 3). In the group of smoking AP patients with the GC genotype, the IL-6 concentration was two-fold higher in comparison with patients from this group with the GG (*p* < 0.0001) and CC (*p* = 0.0154) genotypes (Table 3). 

### 3.3. The Concentration and Activity of SOD Isoenzymes in the Plasma of Non-Smoking and Smoking Healthy Subjects and AP Patients in Terms of rs1800795 in IL6 Gene

**Figure 2:**
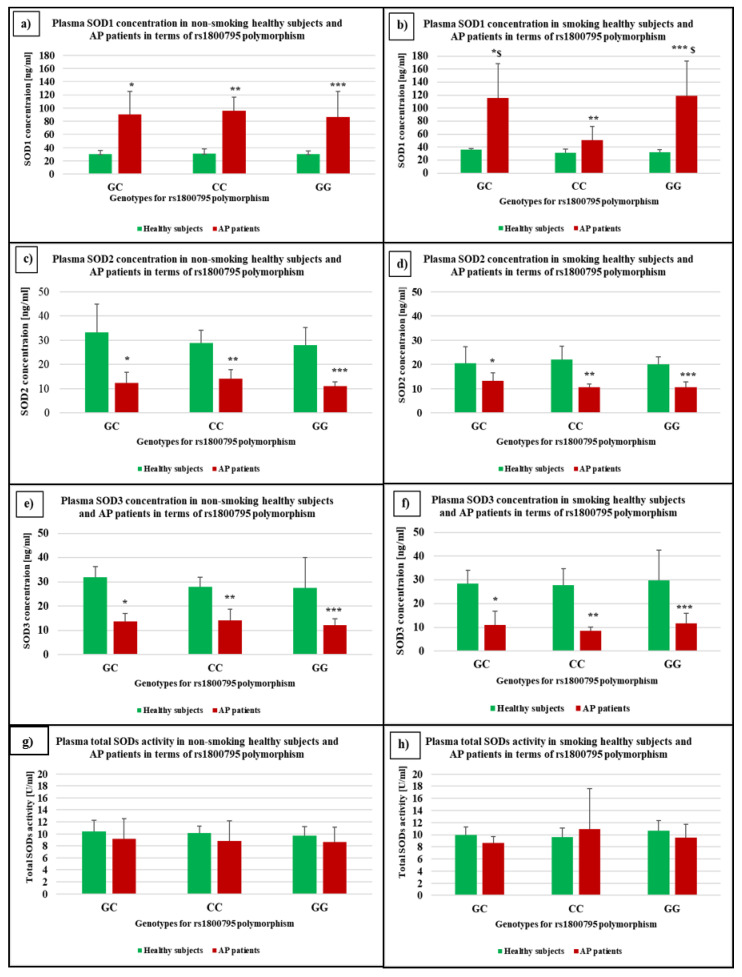
The concentration of plasma SOD1 in the group of (**a**) non-smoking and (**b**) smoking healthy subjects and AP patients; the concentration of plasma SOD2 in the group of (**c**) non-smoking and (**d**) smoking healthy subjects and AP patients; the concentration of plasma SOD3 in the group (**e**) non-smoking and (**f**) smoking healthy subjects and AP patients; the plasma total SOD activity in the group of (**g**) non-smoking and (**h**) smoking healthy subjects and AP patients in terms of rs1800795 in IL6 gene-CRP. * *p* < 0.05—compare to healthy subjects with GC genotype; ** *p* < 0.05—compare to healthy subjects with CC genotype; *** *p* < 0.05—compare to healthy subjects with GG genotype; ^$^ compare to AP patients with CC genotype.

An increase in the SOD1 concentration in the plasma of non-smoking (*p* < 0.0001 for all compared genotypes) and smoking (*p* = 0.0284, *p* = 0.0304, and *p* = 0.0005, respectively) AP patients with the GC, CC, and GG genotypes compared to healthy subjects with corresponding genotypes was shown (Figure 2a,b). In addition, in the plasma of smoking AP patients with the GC and GG genotypes, the SOD1 concentration was significantly higher (*p* = 0.0146 and *p* = 0.0250, respectively) than in patients with the CC genotype (Figure 2b). Furthermore, in the plasma of non-smoking and smoking AP patients with the GC, GG, and GG genotypes, a decrease in the SOD2 concentration (for non-smokers *p* = 0.0143, *p* = 0.0022, and *p* = 0.0050 and for smokers *p* = 0.0356, *p* = 0.0225, and *p* = 0.0317, respectively) was demonstrated in contrast to healthy subjects (Figure 2c,d). A decrease in SOD3 concentration in the plasma of non-smoking and smoking AP patients with the GC, GG, and GG genotypes (*p* < 0.0001 for all genotypes for non-smokers and smokers) compared to healthy subjects was demonstrated (Figure 2e,f). No statistical difference between genotypes in total SOD activity in plasma both in non-smoking and smoking AP patients and healthy subjects was shown (Figure 2g,h). 

### 3.4. The Concentration and Activity of SOD Isoenzymes in the Erythrocytes of Non-Smoking and Smoking Healthy Subjects and AP Patients in Terms of rs1800795 in IL6 Gene

In the erythrocytes of smoking AP patients with the GC genotypes, an increase in the SOD1 concentration compared to the individuals from this group with the GG genotype (*p* = 0.0408) was demonstrated (Figure S2b). Whereas, in erythrocytes of non-smoking (*p* < 0.0001 for all genotypes) and smoking (*p* = 0.0225, *p* = 0.0304, and *p* = 0.0278, respectively) AP patients, SOD2 was significantly higher in comparison with healthy subjects with corresponding genotypes (Figure S2c,d). Moreover, in erythrocytes of non-smoking and smoking AP patients with the GC, CC, and GG genotypes, the total SOD activity was three-fold higher (for non-smokers *p* = 0.0027, *p* = 0.0112, and *p* < 0.0001 and smokers *p* = 0.0027, *p* = 0.0045, and *p* < 0.0001, respectively) than in healthy subjects with corresponding genotypes (Figure S2e,f)

### 3.5. Results for the Analysis of Odds Ratio and Correlation 

No association between AP occurrence and tobacco smoke-exposure in the subjects with GC (OR = 0.8746, *p* = 0.9979), GG (OR = 0.7989, *p* = 0.8545), and CC genotypes (OR = 1.0000, reference) for SNPs 1800795 in the *IL6* gene was observed. Additionally, a positive correlation between IL-6 and plasma SOD1 concentrations (r^2^ = 0.6571, *p* = 0.0374) in the AP patients group with the GC genotype was found in this study.

## 4. Discussion

The pro-antioxidant imbalance may contribute to disturbances in cellular signaling pathways [30,31], which is demonstrated by increased expression of pro-inflammatory cytokines [6]. This study showed more than a 150-fold increase in IL-6 concentration in the AP patients group in comparison with the healthy subjects group, confirming that IL-6 is an essential mediator in the pathophysiology of acute pancreatitis. Hence, it can be a result of an increased inflammatory response accompanying the intensive oxidative stress [32]. SOD isoenzymes are believed to be potential inhibitors of inflammation, which we confirmed in previous study [28]. SOD isoenzymes may be involved in the inflammatory response of the pancreas. We examined the concentrations of SOD isoenzymes and total activity in order to investigate these parameters in the intracellular (erythrocytes) and extracellular environments to compare these two environments and define the roles of SOD in them. SOD1 is an isoenzyme involved in the defense of red blood cells against protein and lipid oxidation, in which structural changes may contribute to anemia or activation of erythropoiesis [28,33]. In addition, based on recent reports, SOD2 may be involved in the defense of red blood cells against their reduced deformability due to oxidative stress [34]. We found increased SOD2 concentration and total SOD activity in the erythrocytes of AP patients, which evidenced that SOD plays a key role in intracellular antioxidative defense [28].

As inflammation elevates, the antioxidant defense system also increases, which we observed as an increase in IL-6 concentration and the SOD1 concentration in the plasma of AP patients. It means that SOD1 may protect against oxidative damage in the cell caused by inflammation. Other studies also confirm that SOD is the important enzymatic component of the antioxidant defense in the organism [28]. In the plasma of patients with acute pancreatitis, a lower concentration of SOD2 was shown, compared to healthy subjects. Based on the above observations, it can be assumed that the reduced values of the concentration of SOD2 may contribute to the reduced elimination of superoxide anions. An impaired neutralization of oxidative stress can result in turn in the intensification of tissue damage and the development of inflammation, which was reflected in increased IL-6 and hs-CRP concentration in the course of acute pancreatitis. SOD2 use can confirm the important role of this isoenzyme in free-radical neutralization, which was reported in an earlier study [35]. In the extracellular environment, SOD3 is the major isoenzyme of SODs [36]; therefore, its concentration in plasma was measured. The reduced concentration of SOD3 in the plasma of AP patients, as shown in our results, compared to healthy subjects, may be associated with elevated superoxide anions production during inflammatory conditions [37].

Based on the literature, it is known that genetic polymorphisms in the gene encoding IL-6 may influence its blood concentration and change the functionality of this protein [17,38]. SNP rs1800795 in the *IL6* gene may be the determinant of differences in personal sensitivity to the inflammatory response and increased oxidative stress [39,40]. The presence of the GC genotype for this polymorphism in the *IL6* gene can be associated with the progression of the inflammatory response in the organism in the course of inflammatory diseases. However, there are no studies about polymorphism in the *IL6* gene and its association with the concentration of SOD isoenzymes and total SOD activity during acute pancreatitis. 

The reports in the literature about the polymorphism rs1800795 in the *IL6* gene are contradictory. One of them presented that the highest concentration of IL-6 was associated with the GG genotype [41]. In another report, no differences in IL-6 concentration were found between different genotypes of this polymorphism [42]. Our study indicates that this SNP in the *IL6* gene was associated with a higher IL-6 concentration in the group of smoking AP patients with the GC genotypes in comparison with patients with the CC and GG genotypes. Additionally, AP patients with the GC genotype for this SNP showed higher SOD1 concentrations in plasma and erythrocytes. The values of SOD1 and IL-6 concentrations are related, which confirmed the positive correlation of these parameters in the plasma of AP patients with the GC genotypes for SNP rs1800795. These results may indicate that SOD1 can play a protective role for cells against oxidative damage caused by inflammation [1,4,5]. 

We did not show the statistically significant difference between genotypes both in SOD2 and SOD3 concentrations and total SOD activity in plasma and erythrocytes of non-smoking and smoking AP patients in terms of examined SNP in the *IL6* gene. Additionally, we did not notice the correlation between IL-6 concentration and concentrations of SOD2 and SOD3 and total SOD activity both in plasma and erythrocytes of non-smoking and smoking AP patients in terms of SNP rs1800795 in the *IL6* gene. This is evidence that this polymorphism in the *IL6* gene did not affect concentrations of SOD2 and SOD3 isoenzymes in the blood. 

In our study, no associations between individual genotypes for SNP rs1800795 in the *IL6* gene and AP risk were shown, confirming the investigation of Chi et al. [43], in which no relationship between the occurrence of this polymorphism and the risk of AP was found. This is also evidenced by the frequency of occurrence of individual genotypes, which were similar in the group of AP patients and healthy subjects. This ultimately proves that the polymorphism rs1800795 in the *IL6* gene is not associated with the occurrence of acute pancreatitis.

On the basis of the obtained results, it can be concluded that the studied polymorphisms may be a valuable diagnostic tool in acute pancreatitis. Moreover, the association of the SOD1 and IL-6 concentrations in the AP patients with the G/C genotype for SNP rs1800795 can indicate that SOD1 may be a target for the development of new therapies. Based on literature reports, the therapeutic potential and physiological importance of SOD isoenzymes is recognized [20]. It is known that SOD was used as a therapeutic agent in diseases associated with increased oxidative stress [44]. It has been shown that SODs can be used to reduce inflammation as well as prevent the proliferation of precancerous cells [44]. Moreover, it has shown a high potential in SOD mimetics (small molecule catalytic antioxidant) in the treatment of diseases associated with the increased oxidative stress [20]. The involvement of SOD mimetics to neutralize O_2_ could prevent infiltration of neutrophils at the site of damage [41]. This property of SOD can be used to inhibit the progression of inflammation in the course of AP. In summary, the GC genotype of rs1800795 polymorphism in the *IL6* gene is associated with a higher concentration of IL-6 and the concentration of SOD1 in plasma and erythrocytes in the group of smoking AP patients compared to patients with the GG and CC genotypes. Correspondingly, as IL-6 concentration increases, SOD1 concentration increases, which may confirm that SOD1 plays a protective role for cells against oxidative damage due to inflammation. 

## 5. Closing Remarks

The homogenous study population and well-defined AP patients and healthy control groups are the advantages of this work. However, the association between the concentration of SOD1 and the genetic variability on inflammation reflecting in the concentration of IL-6 is more complex and, most probably, could be affected by other factors to be discovered in future research. In the literature, there is poor information about the association between SNP rs1800795 in the *IL6* gene and the concentration of SOD1 in the course of AP, which can limit the comparison of our results with other studies. Because of that, this study should be perceived as preliminary, setting up new insights to understand oxidative stress in AP. Moreover, our study was limited by a small number of individuals included in the study population, which was caused by recruitment rules assuming the resemblance of AP patients to healthy individuals in terms of age and anthropometric parameters. Due to the abovementioned drawbacks, our study should be validated by future studies.

## 6. Conclusions

Acute pancreatitis is accompanied by an increased antioxidant defense as evidenced by the increase in plasma SOD1 concentration.

The presence of the GC genotype for SNP rs1800795 in the *SOD1* gene in the group of AP patients is associated with an increase in the concentration of IL-6 and the concentration of SOD1 in the plasma and erythrocytes. 

A positive correlation between plasma SOD1 and IL-6 concentrations in the group of AP patients with the GC genotype confirm that SOD1 may play a protective role for cells against oxidative damage due to inflammation. These results can be considered as preliminary and require confirmation in further studies.

## Figures and Tables

**Table 1:** Clinical characteristics of the patients with AP.

Parameters	AP Patients (*n* = 40)		
	Non-Smokers (*n* = 17)	Smokers (*n* = 23)	*p*
Cotinine (ng/mL)	1.2 ± 0.8	132.4 ± 51.3	**<0.0001**
Lipase (U/L)	791.4 ± 545.5	820.6 ± 540.9	0.5532
α-Amylase (U/L)	548.0 ± 396.5	461.6 ± 343.7	0.7666
ALAT (U/L)	107.5 ± 142.3	80.4 ± 125.4	0.5277
AspAT (U/L)	53.5 ± 45.8	71.3 ± 72.4	0.3037
GGT (U/L)	260.7 ± 297.3	225.8 ± 225.4	0.9794
Bilirubin (total) (mg/dL)	2.2 ±1.8	1.8 ± 2.0	0.6105
Alkaline phosphatase (U/L)	205.5 ± 231.5	149.6 ± 114.6	0.3825
Glucose (mg/dL)	85.4 ± 17.5	89.3 ± 9.8	0.8983

**Table 2:** The concentration of markers of inflammation, concentration of SOD isoenzymes, and total SOD activity in the group of non-smoking and smoking healthy subjects and AP patients.

	Healthy Subjects			AP Patients		
Parameters	Non-Smokers (*n* = 26)	Smokers (*n* = 25)	*p*	Non-Smokers (*n* = 17)	Smokers (*n* = 23)	*p*
IL-6 (pg/mL] (plasma)	0.5 ± 0.3 (0.1; 0.4; 0.6)	0.5 ± 0.2 (0.2; 0.3; 0.8)	0.8012	55.3 ± 27.8 * (31.4; 53.2; 79.2)	82.8 ± 25.7 ** ( 53.5; 92.7; 102.1)	**0.0025**
hs-CRP (mg/dL] (serum)	0.6 ± 0.3 (0.4; 0.5; 0.7)	0.5 ± 0.2 (0.3; 0.4; 0.6)	0.2585	126.2 ± 19.8 * (106.5; 131.6; 134.7)	135.2 ± 40.8 ** (121.0; 131.7; 158.3)	0.5828
SOD1 (ng/mL) (plasma)	30.1 ± 4.8 (26.5; 29.2; 33.6)	33.7 ± 5.4 (28.7; 34.0; 37.2)	0.0847	81.7 ± 21.2 * (64.4; 86.9; 96.9)	85.5 ± 26.7 ** (69.4; 79.0; 90.4)	0.7276
SOD1 (ng/mg Hb) (erythrocyte lysate)	5.8 ± 1.9 (4.5; 5.0; 6.8)	5.8 ± 2.0 (4.6; 5.1; 5.8)	0.2799	5.0 ± 2.0 (3.9; 4.9; 6.1)	5.6 ± 2.1 (4.6; 5.3; 6.6)	0.4850
SOD2 (ng/mL) (plasma)	28.6 ± 9.2 (22.1; 24.5; 37.1)	23.9 ± 7.4 (17.7; 22.0; 32.1)	0.4306	11.8 ± 2.4 * (9.6; 11.5; 14.4)	11.6 ± 3.4 ** (9.5; 10.3; 11.0)	0.9220
SOD2 (ng/mg Hb) (erythrocyte lysate)	2.2 ± 1.0 (1.5; 1.6; 2.5)	2.0 ± 0.9 (1.5; 2.0; 2.7)	0.3053	9.5 ± 3.9 * (5.3; 9.6; 14.3)	9.4 ± 2.0 ** (6.1; 9.1; 7.0)	0.6136
SOD3 (ng/mL) (plasma)	25.8 ± 10.1 (15.7; 29.3; 30.6)	25.7 ± 11.4 (17.8; 20.7; 38.8)	0.9975	10.9 ± 3.3 * (7.6; 9.5; 12.2)	10.9 ± 2.0 ** (7.0; 8.9; 9.6)	0.1104
Total SODs (U/mL) (plasma)	10.1 ± 1.3 (9.1; 10.3; 11.1)	10.1 ± 1.5 (8.9; 10.1; 11.3)	0.8814	8.9 ± 2.9 (6.5; 8.4; 10.8)	9.1 ± 2.6 (7.2; 8.2; 10.8)	0.4783
Total SODs (U/g Hb) (erythrocyte lysate)	157.4 ± 60.0 (114.3; 142.2; 194.7)	141.4 ± 48.8 (103.7; 140.0; 176.6)	0.3504	416.1 ± 81.2 * (357.1; 401.1; 440.8)	429.8 ± 77.9 ** (377.2; 403.9; 476.4)	0.6613

* *p* < 0.05—compare to non-smoking healthy subjects. ** *p* < 0.05—compare to smoking healthy subjects.

**Table 3:** The concentration of IL-6 and hs-CRP in the group of non-smoking and smoking healthy subjects and AP patients in terms of rs1800795 in *IL6* gene.

Parameters	Non-Smokers					
	Healthy Subjects (*n* = 26)			AP Patients (*n* = 23)		
	GC (*n* = 10)	CC (*n* = 3)	GG (*n* = 13)	GC (*n* = 6)	CC (*n* = 5)	GG (*n* = 12)
IL-6 (pg/mL) (plasma)	0.6 ± 0.3 (0.4; 0.6; 0.7)	0.2 ± 0.1 (0.1; 0.2; 0.2)	0.2 ± 0.1 (0.1; 0.1; 0.3)	49.6 ± 27.3 * (24.5; 49.6; 74.6)	36.0 ± 11.4 ** (24.9; 35.4; 47.7)	48.9 ± 30.2 *** (30.9; 31.9; 83.8)
hs-CRP (mg/dL) (plasma)	0.6 ± 0.2 (0.4; 0.5; 0.8)	0.6 ± 0.2 (0.4; 0.5; 0.6)	0.5 ± 0.1 (0.4; 0.4; 0.6)	133.1 ± 2.2 * (131.6; 133.1; 134.7)	140.9 ± 48.4 ** (101.7; 151.4; 180.0)	123.6 ± 27.2 *** (101.8; 120.1; 145.4)
**Parameters**	**Smokers**					
	**Healthy Subjects (*n* = 25)**			**AP Patients (*n* = 17)**		
	**GC** **(*n* = 5)**	**CC** **(*n* = 10)**	**GG** **(*n* = 10)**	**GC** **(*n* = 7)**	**CC** **(*n* = 3)**	**GG** **(*n* = 7)**
IL-6 (pg/mL) (plasma)	0.3 ± 0.1 (0.2; 0.3; 0.3)	0.3 ± 0.1 (0.1; 0.3; 0.4)	0.5 ± 0.3 (0.3; 0.5; 0.8)	97.4 ± 6.6 *^,$,‡^ (92.7; 97.4; 102.1)	44.9 ± 12.6 ** (25.8; 35.4; 46.7)	38.8 ± 20.9 *** (24.0; 38.8; 53.5)
hs-CRP (mg/dL) (plasma)	0.6 ± 0.2 (0.2; 0.6; 0.9)	0.5 ± 0.2 (0.3; 0.5; 0.8)	0.4 ± 0.1 (0.3; 0.4; 0.4)	110.0 ± 39.7 * (105.4; 130.9; 132.6)	169.8 ± 41.5 ** (124.6; 178.7; 206.2)	144.4 ± 38.0 *** (111.4; 134.7; 184.5)

* *p* < 0.05—compare to healthy subjects with GC genotype; ** *p* < 0.05—compare to healthy subjects with CC genotype; *** *p* < 0.05—compare to healthy subjects with GG genotype; ^$^ *p* < 0.05—compare to AP patients with CC genotype; ^‡^ *p* < 0.05—compare to AP patients with GG genotype.

## Supplementary Materials

The following are available online at https://www.mdpi.com/article/10.3390/genes13020290/s1, Figure S1: Example of electropherogram for rs1800795 in *IL6* gene. Figure S2: The concentration of SOD1 (a,b), SOD2 (c,d) and total SODs activity (e,f) in erythrocytes of non-smoking and smoking healthy subjects and AP patients in terms of rs1800795 in IL6 gene.
