# Supplementary material for: Association of Genetic Variants in IL6 Gene (rs1800795) with the Concentration of Inflammatory Markers (IL-6, hs-CRP) and Superoxide Dismutase in the Blood of Patients with Acute Pancreatitis—Preliminary Findings
Source: Genes (Basel). 2022 Feb 1;13(2):290. doi: 10.3390/genes13020290 (PMC8872489; doi:10.3390/genes13020290)
Supplement: Supplementary file 1 [file genes-13-00290-s001.zip › genes-1555338-supplementary.pdf]

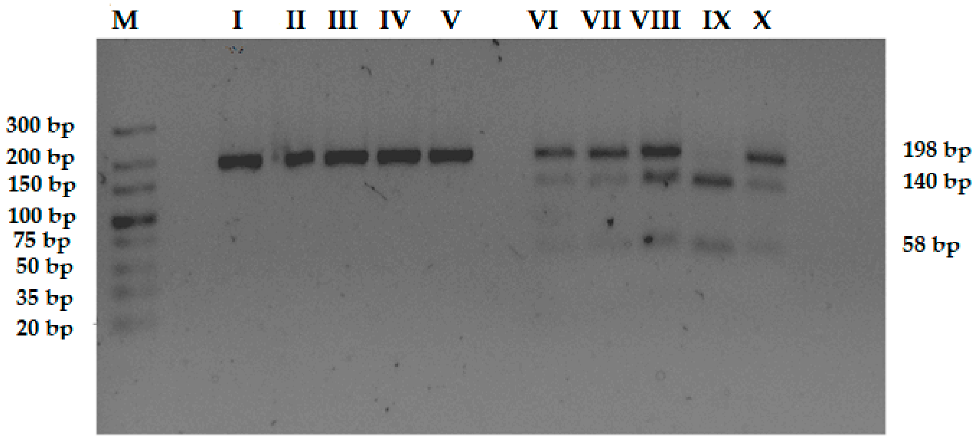

Figure S1. Example of electropherogram for rs1800795 in *IL6* gene. M—marker ladder (20 - 300 bp); I, II, III, IV, V–GG genotype (198 bp fragment); VI, VII, VIII, X – GC genotype (198, 140, 58 bp fragments), IX – CC genotype (140 bp and 58 bp fragments). Numbers are in base pair (bp).

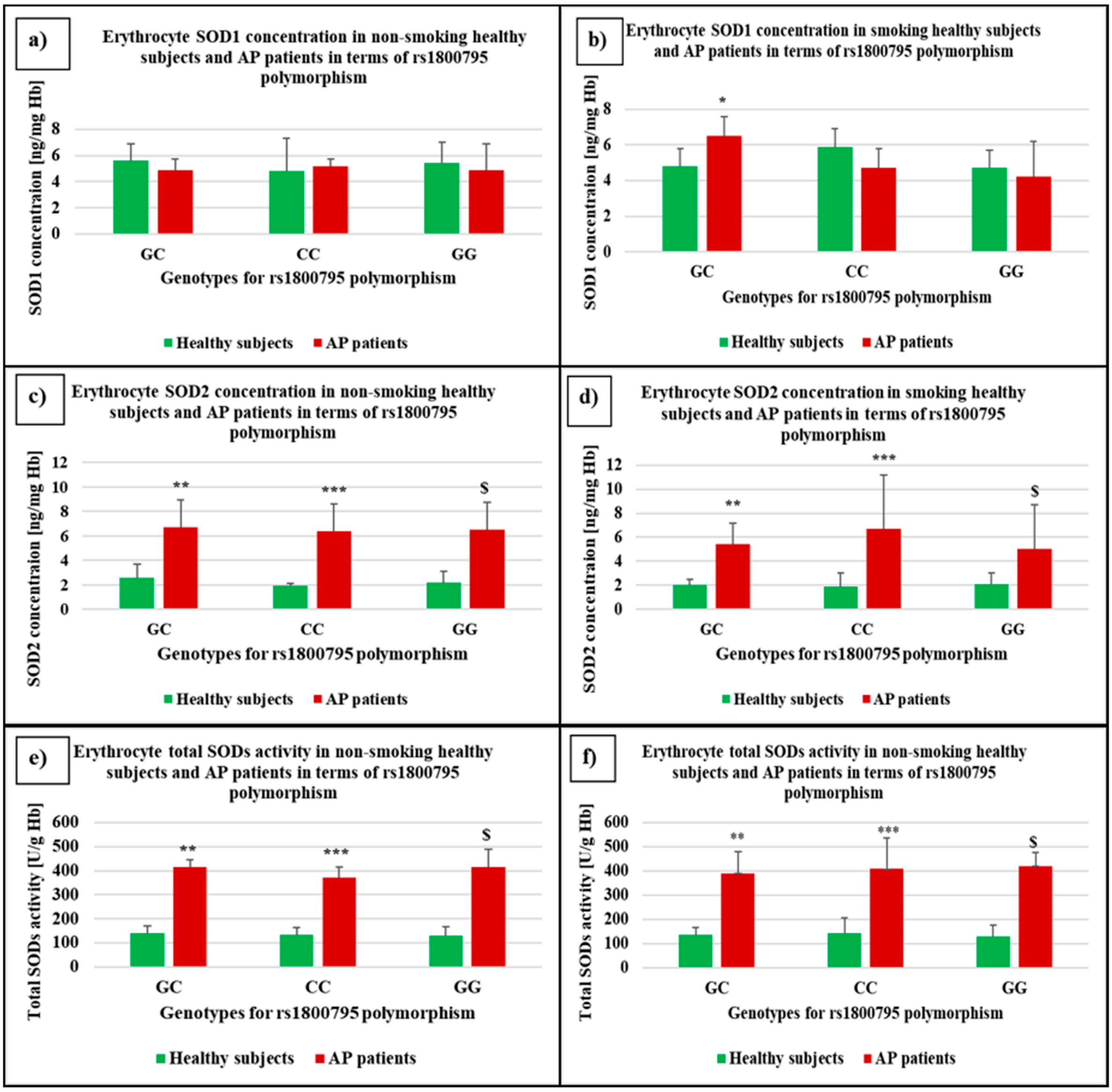

Figure S2. The concentration of SOD1 (a, b), SOD2 (c, d) and total SODs activity (e, f) in erythrocytes of non-smoking and smoking healthy subjects and AP patients in terms of rs1800795 in IL6 gene. * compare to AP patients with GG genotype, ** $p < 0.05$ – compare to healthy subjects with GC genotype, *** $p < 0.05$ – compare to healthy subjects with CC genotype, $\$$ $p < 0.05$ – compare to healthy subjects with GG genotype.
